# Supplementary material for: Intracoronary Injection of Autologous CD34+ Cells Improves One-Year Left Ventricular Systolic Function in Patients with Diffuse Coronary Artery Disease and Preserved Cardiac Performance—A Randomized, Open-Label, Controlled Phase II Clinical Trial
Source: J Clin Med. 2020 Apr 7;9(4):1043. doi: 10.3390/jcm9041043 (PMC7231253; doi:10.3390/jcm9041043)
Supplement: Supplementary file 1 [file jcm-09-01043-s001.pdf]

Supplementary Table 1. Comparison of echocardiographic parameters between two groups at baseline and 12 months

Variables	Study group (n=25)	Control group (n=27)	p-value
Baseline echocardiography			
LA diameter, mm	43.68±5.34	44.81±8.95	0.585
Grade of diastolic dysfunction	1.36±0.70	1.32±0.69	0.840
E/A ratio	1.05±0.74	1.05±0.51	0.989
Average E/E′ ratio	12.70±5.07	13.32±3.97	0.639
2D LVEF, %	51.38±11.61	50.96±13.54	0.907
LV end-diastolic diameter, mm	54.22±8.23	56.14±11.61	0.499
LV end-systolic diameter, mm	40.00±8.94	41.61±12.17	0.592
3D LVEF, %	50.26±12.93	51.92±12.81	0.644
LV end-diastolic volume, mL	79.65±24.68	79.79±30.78	0.812
LV end-systolic volume, mL	41.07±20.33	37.51±21.15	0.540
Systolic dyssynchrony index, %	10.85±6.57	8.79±5.88	0.270
12-month echocardiography			
LA diameter, mm	43.18±7.29	43.98±8.51	0.718
Grade of diastolic dysfunction	1.12±0.60	1.27±0.72	0.428
E/A ratio	0.90±0.43	0.95±0.65	0.738
Average E/E′ ratio	11.99±3.45	13.23±6.93	0.430
2D LVEF, %	55.12±12.65	52.21±14.61	0.447
LV end-diastolic diameter, mm	56.12±6.36	55.90±8.36	0.916
LV end-systolic diameter, mm	39.69±8.00	39.37±11.79	0.911
3D LVEF, %	54.88±12.90	50.98±13.61	0.295
LV end-diastolic volume, mL	76.17±27.29	70.71±30.32	0.503
LV end-systolic volume, mL	37.22±23.07	35.97±23.46	0.848
Systolic dyssynchrony index, %	7.18±5.92	8.53±6.20	0.433

Abbreviation: LA, left atrial; E/A ratio, the ratio of the early (E) to late (A) ventricular filling velocities; E/E′ ratio, the ratio of mitral peak velocity of early filling (E) to early diastolic mitral annular velocity (E'); 2D or 3D, 2- or 3-dimentional echocardiography; LVEF, left ventricular ejection fraction; LV, left ventricular.

Data are expressed as mean ± standard deviation.

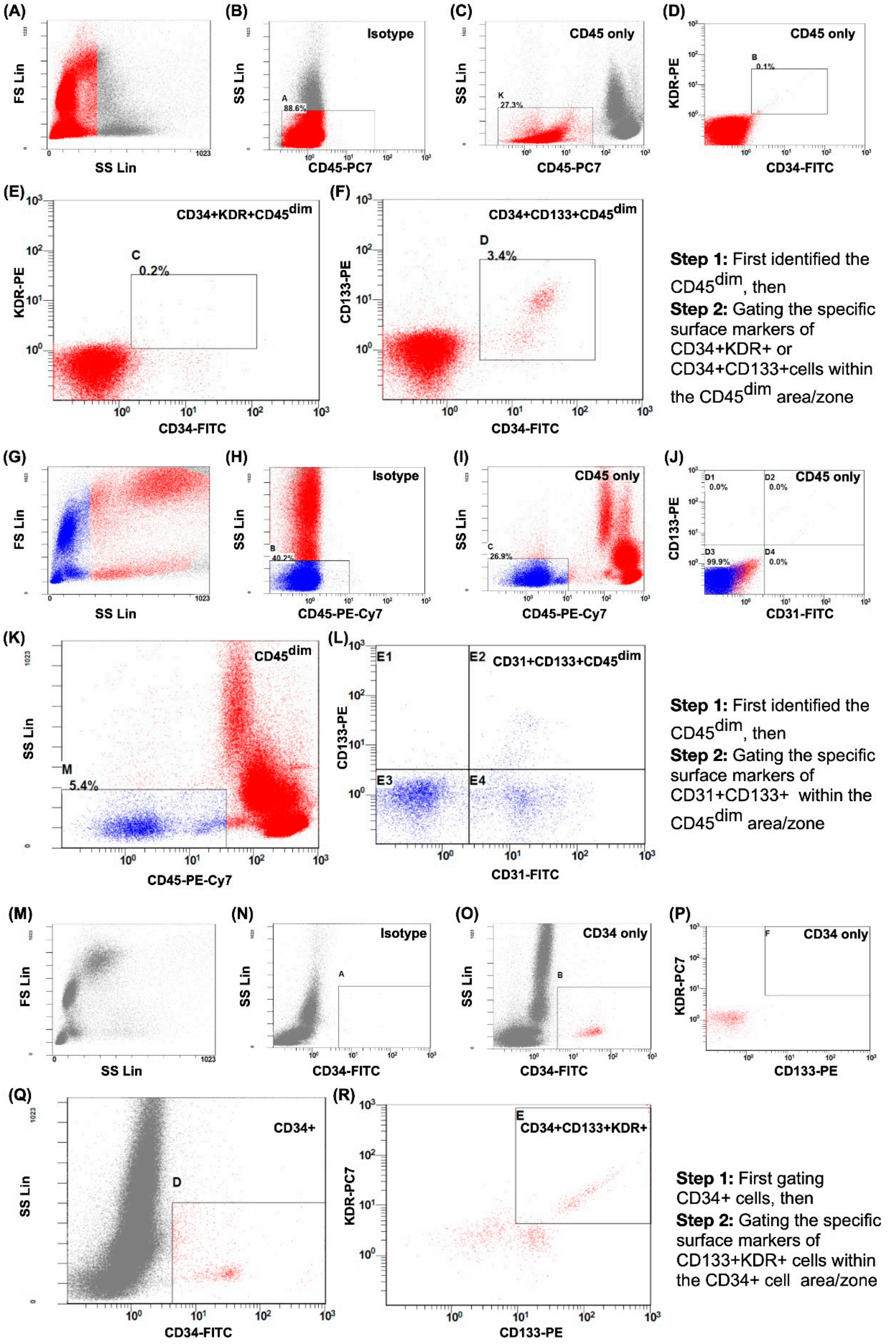

Supplemental Figure 1 (A to R). Procedure for gating individual EPC surface makers. A) to **R)** the representative dot plots show the procedures how we gated the individual EPC surface markers by CXP Analysis software (Beckman Coulter).

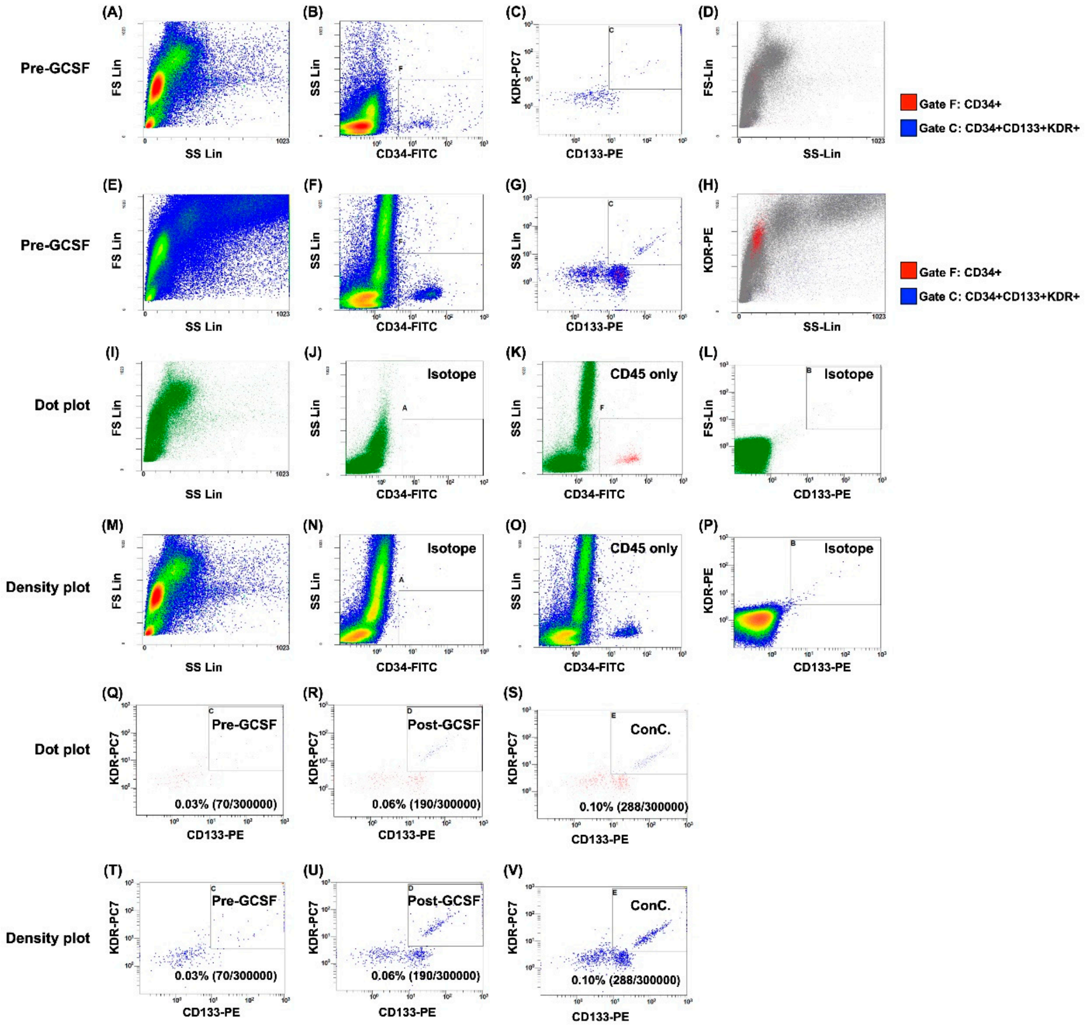

Supplemental Figure 2 (A to H). Illustrating the presentation of the backing gate for EPC surface maker.

A) to **H)** Showing the flow cytometric analyses of the backing gate by CXP Analysis software (Beckman Coulter) in Pre-GCSF and Post-GCSF groups.
